# FreqDyn-YOLO: A High-Performance Multi-Scale Feature Fusion Algorithm for Detecting Plastic Film Residues in Farmland

**DOI:** 10.3390/s25164888

**Published:** 2025-08-08

**Authors:** Mingyang Zhang, Jianjie Zhang, Yihang Peng, Yi Wang

**Affiliations:** 1 College of Software, Xinjiang University, Urumqi 830091, China; 107552305021@stu.xju.edu.cn (M.Z.); 107552404905@stu.xju.edu.cn (Y.W.); 2 College of Mechanical Engineering, Xinjiang University, Urumqi 830017, China; 107552304306@stu.xju.edu.cn

**Keywords:** YOLO11, residual film, FreqFTA, HPMSFPN, DCNv4

## Abstract

Plastic mulch technology plays an important role in increasing agricultural productivity and economic returns. However, residual mulch remaining in agricultural fields poses significant challenges to both crop production and environmental sustainability. Effective recovery and recycling of residual plastic mulch requires accurate detection and identification of mulch fragments, which presents a substantial technical challenge. The detection of residual plastic film is complicated by several factors: the visual similarity between residual film fragments and soil in terms of color and texture, as well as the irregular shapes and variable sizes of the target objects. To address these challenges, this study develops FreqDyn-YOLO, a detection model for residual film identification in agricultural environments based on the YOLO11 architecture. The proposed methodology introduces three main technical contributions. First, a Frequency-C3k2 (FreqC3) feature extraction module is implemented, which employs a Frequency Feature Transposed Attention (FreqFTA) mechanism to improve discrimination between residual film and soil backgrounds. Second, a High-Performance Multi-Scale Feature Pyramid Network (HPMSFPN) is developed to enable effective cross-layer feature fusion, enhancing detection performance across different target scales. Third, a Dynamic Detection Head With DCNv4 (DWD4) is introduced to improve the model’s ability to adapt to varying film morphologies while maintaining computational efficiency. Experimental findings on a self-developed agricultural field residual film dataset confirm that FreqDyn-YOLO outperforms the baseline approach, achieving improvements of 5.37%, 1.97%, and 2.96% in mAP50, precision, and recall, respectively. The model also demonstrates superior performance compared to other recent detection methods. This work provides a technical foundation for precise residual film identification in agricultural applications and shows promise for integration into automated recovery systems.

## 1. Introduction

Plastic mulching represents a significant agricultural practice in contemporary farming systems, wherein plastic films are applied to soil surfaces to accomplish diverse agronomic functions, including soil moisture conservation, thermal regulation, weed control, pathogen mitigation, crop quality enhancement, and yield optimization [1,2]. This technology has evolved into one of the most extensively adopted cultivation techniques within China’s agricultural sector. Nevertheless, the progressive expansion of plastic mulching applications and the consequent annual increase in coverage area have accentuated inherent limitations of this practice, including film fragility, poor biodegradability, and recovery complexities, thereby exacerbating the accumulation of plastic residues in agricultural soils [3,4]. The presence of plastic film fragments compromises soil structural integrity, diminishes water retention capacity and nutrient availability, and induces soil compaction, consequently impeding root system development and normal plant growth processes, thus presenting substantial challenges to sustainable agricultural practices [4,5].

The escalating severity of plastic film residue contamination makes the efficient retrieval of agricultural plastic waste critical for environmental stewardship in farming systems [6]. Although conventional manual collection methods demonstrate superior recovery quality, they have substantial limitations, including intensive labor requirements, diminished operational efficiency, and elevated costs, rendering them impractical for the large-scale remediation of farmland plastic residues [7,8]. While mechanized retrieval technologies can substantially enhance operational productivity, current plastic film recovery machinery exhibits constrained performance in detecting and capturing small-scale film fragments, with incomplete recovery remaining a persistent challenge [9,10,11]. Consequently, the development of intelligent plastic film recovery systems capable of achieving precise detection and spatial localization of residual materials has emerged as a pivotal technological strategy for improving recovery efficiency and mitigating agricultural plastic pollution [12].

During the initial phases of agricultural film detection research in farmland environments, traditional computer vision methodologies constituted the predominant approach [13,14,15]. These techniques integrated feature extraction with machine learning classification algorithms to facilitate target recognition [16,17]. In early technological developments, Jiang et al. [18] introduced an innovative framework combining histogram threshold segmentation with edge detection algorithms and region-filling techniques to achieve effective separation of residual film imagery. Nevertheless, this methodology resulted in the partial loss of residual film information, consequently increasing localization inaccuracies. With the rapid progression of remote sensing technologies, Zhu et al. [19] utilized unmanned aerial vehicle (UAV) remote sensing platforms to acquire agricultural field imagery [20]. Through the implementation of an integrated algorithm incorporating morphological processing and area threshold segmentation, they successfully extracted spatial distribution data of plastic mulch-covered regions. Fu et al. [21] s ubsequently addressed the constraints of single-source data limitations by developing a real-time extraction framework for multi-source remote sensing data fusion, effectively resolving the challenges associated with plastic mulch identification in topographically complex agricultural environments.

The proliferation of deep learning technologies has facilitated the extensive implementation of deep learning-based computer vision algorithms in agricultural film residue detection, effectively superseding conventional feature selection methodologies [22]. Through the utilization of sophisticated visual systems and machine learning algorithms, precise identification and localization of agricultural film residues can be achieved, substantially enhancing both efficiency and quality of residue recovery operations [23]. Deep learning-based computer vision detection approaches demonstrate superior capability in learning complex feature representations, thereby markedly improving detection accuracy. Prevalent object detection algorithms encompass the YOLO series, SSD, EfficientDet, R-CNN, Mask R-CNN, and Faster R-CNN frameworks [24]. Zhang et al. [25] i mplemented an enhanced Faster R-CNN convolutional neural network for residual film recognition and detection in agricultural settings, successfully fulfilling technical specifications for residual film identification. Zhou et al. [26] i ntroduced the MFFM Faster R-CNN algorithm, which addresses multi-scale variation challenges in residual film detection through a multi-feature fusion mechanism, thereby augmenting feature extraction capabilities. However, this methodology presents limitations including complex training procedures, substantial redundant information in adjacent detection windows, and numerous invalid regions, resulting in elevated computational requirements and relatively diminished detection velocities [27]. Chen et al. [28] p roposed the YOLOv7-WFD algorithm, which substantially enhances the model’s target feature extraction capabilities. Nevertheless, due to the heterogeneous and complex nature of agricultural waste materials, detection accuracy for residual film targets requires further optimization.

In conclusion, existing agricultural film identification and detection technologies predominantly concentrate on recognizing intact films, which possess advantageous characteristics, including extensive coverage areas, distinctive geometric properties, and pronounced spectral signatures. However, research investigating the detection of residual films in post-harvest agricultural environments remains limited, making it difficult to detect residual film. Following prolonged exposure to environmental factors, such as wind and precipitation, residual mulch materials in farmland exhibit negligible differences from bare soil in terms of textural, morphological, and spectral characteristics, while simultaneously presenting complex attributes including diminutive size, irregular morphology, and stochastic spatial distribution [29]. Conventional detection methodologies demonstrate limited efficacy in addressing these challenges [30]. To resolve these aforementioned limitations, this study proposes a multi-scale feature fusion algorithm for residual mulch detection, specifically designed to address the detection of fragmented residual mulch materials. This approach presents substantial implications for the recovery and reutilization of agricultural plastic waste. The principal contributions of this work include the following:

1. To validate the detection performance of the proposed model, this study developed a detection dataset for residual film using residual film images collected from natural farmland environments, with manual annotation of the residual film targets.
2. A Frequency-C3k2 (FreqC3) feature extraction module was proposed, and the Frequency Feature Transposed Attention (FreqFTA) mechanism was designed to transpose an attention calculation from the spatial dimension to the channel dimension. Through cross-channel information interaction and the frequency gating mechanism of the Dual-Frequency Aggregation Feedforward Network (DFFN), the discriminative ability between residual film and soil background and the perception ability of residual film surface texture features were significantly enhanced.
3. A High-Performance Multi-Scale Feature Pyramid Network (HPMSFPN) using a cross-level feature integration mechanism is proposed, through an efficient multi-scale feature convolution module (EMFC) and a high-performance upsample convolution module (HPUC), which achieves feature fusion between different scales, effectively overcoming the limitations of traditional PAN-FPN in small-object detection.
4. A Dynamic Detection Head With DCNv4 (DWD4) is designed, integrating multi-head self-attention mechanisms, spatial position perception, and task-oriented channel perception. It uses DCNv4 to replace the traditional DCNv2, significantly improving the model’s dynamic adaptability and computational efficiency.

## 2. Dataset Construction

### 2.1. Data Collection

**Figure 1:**
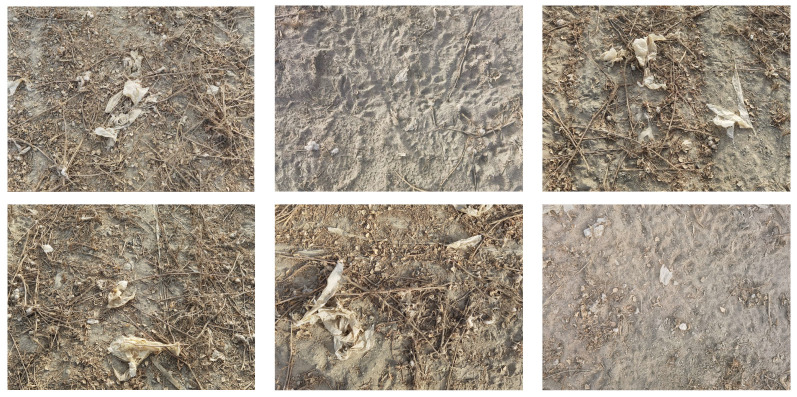
Agricultural residual film image examples.

This study conducted agricultural film residue imagery collection within the cotton cultivation region of Yuli County, Bayingolin Mongol Autonomous Prefecture, Xinjiang. Our investigation focused on plastic film waste remaining across farmland following mechanical operations using an 11JCM-300 plastic film recovery device (manufactured by Dezhou Tianda Runfeng Agricultural Machinery Sales Co., Ltd., Dezhou, Shandong Province, China). Photography was performed using a Huawei Mate 40 Pro smartphone (manufactured by Huawei Technologies Co., Ltd. in Shenzhen, China) at a 4092 × 3072 pixel resolution. Throughout image acquisition, camera positioning remained horizontal, with shooting elevation maintained between 80 and 100 centimeters above ground level. After screening out images with blurring, shaking, or high similarity, a dataset of 1900 residual film images was established. This dataset includes residual film images captured from various angles, under different lighting conditions (ranging from morning to late afternoon hours between 9:00 a.m. and 5:00 p.m.), various weather conditions (sunny, overcast, and partially cloudy days), different soil classes (sandy loam and clay loam soils characteristic of Xinjiang cotton regions), and in various soil moisture conditions, effectively ensuring the diversity of the image samples. Figure 1 shows examples of residual film images captured under different environmental conditions.

### 2.2. Data Annotation

**Figure 2:**
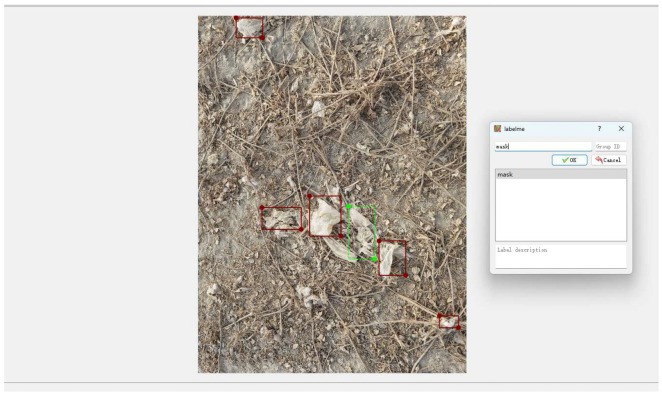
Agricultural residual film annotation.

Agricultural residual film dataset training depends closely on the positional and classification information of plastic films within images, necessitating annotation of these agricultural film samples, as illustrated in Figure 2. In this study, the annotation software Labelme 5.5.0 was used to annotate each residual film image individually, with each annotated residual film target being assigned a mask label. The application saves annotated residual plastic film boundary coordinates within individual JSON documents. Upon completion of all agricultural film image labeling, this collection was partitioned across training, testing, and validation subsets using an 8:1:1 distribution [31], producing 1520 training samples, 190 testing samples, and 190 validation samples. Subsequently, a conversion utility was developed to transform annotation details from sample JSON documents into the TXT format, which were then incorporated into respective subset label directories. Dataset organization followed the Ultralytics YOLO standard [32].

### 2.3. Data Augmentation

**Figure 3:**
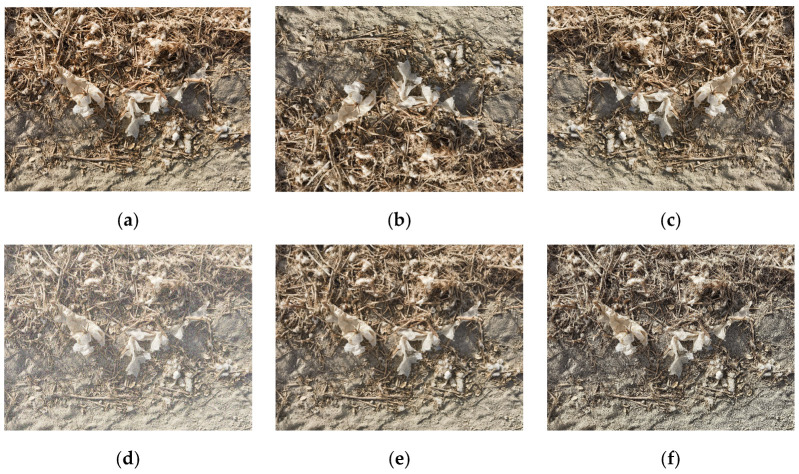
Examples of different augmentation effects. (**a**) Original image. (**b**) Vertical flip. (**c**) Horizontal flip. (**d**) Gaussian noise. (**e**) Brightness and contrast adjustment. (**f**) Sharpening enhancement.

To improve the effectiveness of residual film feature extraction, enhance model generalization performance, and reduce manual annotation costs, this study implemented enrichment and balancing processing on the residual film image dataset. During the training phase, an online data augmentation strategy was used to expand the annotated residual film image dataset, specifically including brightness and contrast adjustment (brightness variation: ±10%, contrast variation: ±10%), sharpening enhancement (kernel intensity: 5.0), vertical flip transformation (probability: 50%), horizontal flip transformation (probability: 50%), and Gaussian noise addition (standard deviation: 1–3), among other diversified data processing techniques. The corresponding augmentation outcomes are illustrated in Figure 3.

The online data augmentation strategy employed in this study generates augmented samples in real time during training, unlike conventional offline methods that pre-process and store samples. The implementation operates through the training framework’s built-in pipeline, where probability-based parameters determine which operations are applied to each image. In this study, we set this probability to 0.5, representing that each image has a 0.5 probability of undergoing augmentation during processing. The system randomly applies various techniques, including geometric transformations, mosaic and mixup operations, and noise injection, while automatically transforming corresponding label coordinates to maintain the annotation accuracy. This approach ensures that the model encounters different augmented variants across training epochs, maximizing data diversity without additional storage requirements and effectively preventing overfitting while enhancing model generalization capabilities.

## 3. Method

### 3.1. YOLO11

**Figure 4:**
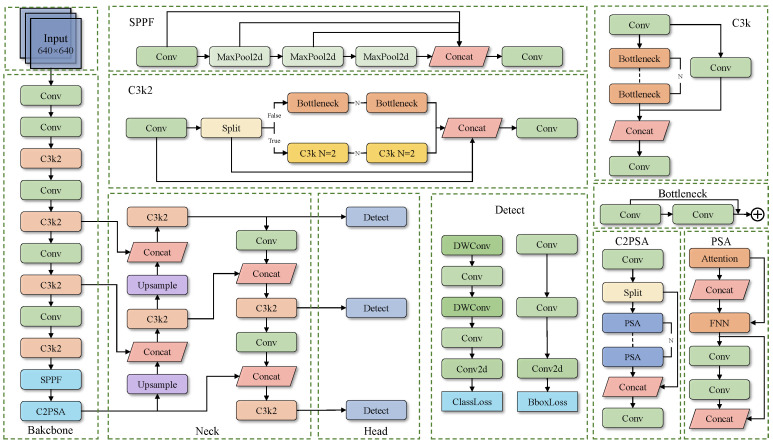
Structure of the YOLO11 model.

The YOLO11 [33] builds upon the successful experience of the previous YOLO series, adopting more efficient feature extraction and multi-scale feature fusion strategies [34]. As illustrated in Figure 4, YOLO11 consists of two main components: the backbone network and the neck network. The backbone network is responsible for extracting multi-level features from the input image. It starts from the input end, with the lower-level CBS (Convolution-BatchNorm-SiLU) module capturing basic texture and edge information, the C3K2 module extracting feature information at different scales to enhance the network’s feature extraction capabilities, the SPPF spatial pyramid pooling module being used to expand the receptive field, and the C2PSA module introducing a spatial attention mechanism, enabling the network to focus on the feature information in the image. The neck network adopts a feature pyramid structure, achieving effective fusion of cross-layer features through upsampling (Upsample) modules and Concat feature concatenation operations. Finally, three detection heads (Head) output prediction results at different resolutions, targeting large, medium, and small objects. YOLO11 inherits the efficient and real-time characteristics of the YOLO series. Through optimized network structure design, it achieves higher detection accuracy while maintaining low computational complexity. Especially through improved modules such as C3K2 and C2PSA, it demonstrates stronger object detection performance in complex scenes. In summary, considering that a good model should have high accuracy, be lightweight, and present real-time advantages, we selected the YOLO11s model as the base model for improvement in this paper.

### 3.2. FreqDyn-YOLO

**Figure 5:**
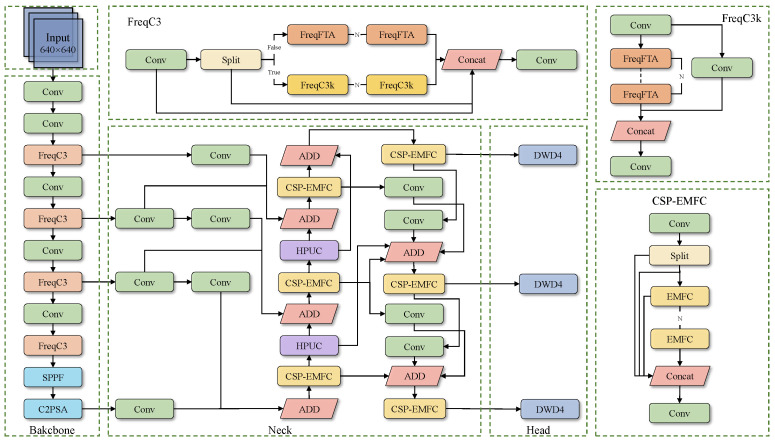
Structure of the FreqDyn-YOLO model.

To address the issue of detecting agricultural film residues in farmland, we designed a new network architecture based on YOLO11: FreqDyn-YOLO. The model architecture is illustrated in Figure 5, with the following specific improvements: First, we improved the feature extraction module C3k2 in the Backbone, replacing it with the FreqC3 module proposed in this paper. We replaced the Bottleneck structure in the original C3k2 module with the Frequency Feature Transposed Attention proposed in this paper. The core of this mechanism lies in transposing the attention calculation from the spatial dimension to the channel dimension, modeling the dependency relationships between different feature channels through cross-channel information interactions, thereby effectively enhancing the discriminative ability between the residual film and soil background. FreqFTA adopts a serial two-stage processing architecture, combining residual connections and normalization, and achieves synergistic optimization of spatial projection and channel projection through a multi-head channel attention mechanism. Additionally, it introduces the Dual-Frequency Aggregation Feedforward Network (DFFN), which uses a frequency gating mechanism [35] to selectively fuse frequency-domain information, significantly enhancing the model’s ability to perceive surface texture features and edge information of residual films. Secondly, a High-Performance Multi-Scale Feature Pyramid Network (HPMSFPN) was constructed to overcome the limitations of traditional PAN-FPN [36] in small-object detection. HPMSFPN adopts a cross-layer feature integration mechanism to effectively combine deep semantic representations with shallow detail characteristics and high-resolution surface-layer features, achieving a dual mechanism of same-scale feature fusion and cross-scale different-resolution layer feature fusion. The network incorporates an efficient multi-scale feature convolution module (EMFC) and a high-performance upsample convolution module (HPUC). The former draws inspiration from the inverted residual module architecture, utilizing multi-scale deep convolution and channel mixing to acquire contextual information across different scales. At the same time, we use EMFC in a cross-stage partial (CSP) structure to replace the original Bottleneck, resulting in the CSP-EMFC module. The latter employs an efficient upconvolution block to achieve progressive upsampling, thereby promoting the fusion of cross-level feature information. Finally, the Dynamic Detect Head With DCNv4 (DWD4) was designed to address the detection challenges posed by the diversity of residual film shapes. This detection head integrates multi-head self-attention mechanisms, spatial position awareness, and task-oriented channel awareness, comprising three core modules: scale-aware attention, spatial-aware attention, and task-aware attention. The key innovation lies in replacing the traditional DCNv2 with DCNv4, which significantly enhances the network’s dynamic adaptability by removing the softmax normalization operation, introducing an adaptive aggregation window, and implementing a dynamic unconstrained weighting strategy. DCNv4 optimizes memory access patterns using instruction-level kernel analysis technology, effectively reducing redundant operations and achieving a significant improvement in computational efficiency.

### 3.3. Frequency-C3k2 (FreqC3)

The original YOLO11 network uses the Bottleneck structure in both the True and False branches of the C3k2 module. The Bottleneck structure extracts hierarchical feature representations from agricultural film residue images through multiple layers of convolution operations, including visual features such as edges, textures, and shapes. However, in agricultural film residue detection tasks, the visual features of the residues and the soil background are highly similar. The Bottleneck structure’s feature extraction mechanism, based on local convolutional kernels, cannot establish long-range spatial dependencies, making it difficult to utilize broader contextual information to enhance the discriminative power between residues and the background. This results in the Bottleneck structure’s insufficient ability to distinguish features within its local receptive field. Additionally, the fixed convolution kernel size of the Bottleneck structure cannot adapt to the multi-scale variations in residual film texture, resulting in inconsistent performance when processing residual films with different degrees of weathering, and thus insufficient depth in texture feature extraction. Residual film fragments in farmland are typically small in size and irregularly distributed. The standard Bottleneck structure is prone to information loss when processing small objects, thereby limiting the model’s ability to detect small objects.

**Figure 6:**
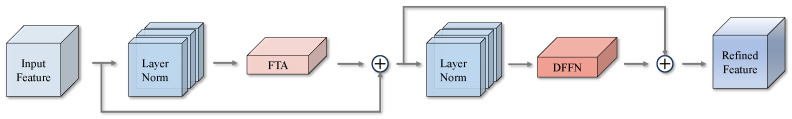
Structure of Frequency Feature Transposed Attention (FreqFTA).

Therefore, we have redesigned a FreqC3 module by enhancing the Bottleneck structure within the C3k2 module with FreqFTA (Frequency Feature Transposed Attention). The fundamental concept of FreqFTA centers on the incorporation of Feature Transposed Attention (FTA), which transposes attention computations from spatial dimensions to channel dimensions, thereby facilitating superior modeling of interdependencies among distinct feature channels and reinforcing semantic associations across channels. This mechanism effectively differentiates visual characteristics between film residues and soil backgrounds. Through cross-channel information interactions, the model’s perceptual capabilities for film residue edge textures are substantially enhanced. Concurrently, we incorporate a DFFN (Dual-Feature Fusion Network) mechanism within FreqFTA, which leverages a gating mechanism in the FrequencyGate to achieve selective fusion of frequency-domain information, enabling adaptive selection of film residue features while effectively extracting and enhancing surface texture characteristics and edge information of film residues. Consequently, through the synergistic operation of these aforementioned mechanisms, FreqFTA significantly augments the model’s feature representation capabilities and detection accuracy for film residue targets in agricultural field detection applications. The specific process of FreqFTA is shown in Figure 6.

The FreqFTA module adopts a serial connection two-stage processing architecture, combining residual links and layer normalization, as shown in Equation (1):

(1) $$Y=F_{FreqFTA}\left(X\right)=T_{reshape}\left(F_{DFFN}\left(F_{FTA}\left(T_{reshape}\left(X\right)\right)\right)\right),$$

where $\tau_{reshape}=R^{B\times C\times H\times W}\to R^{B\times N\times C}$, representing a dimension transformation operation applied to the input X. N = H × W denotes the total number of spatial positions.

**Figure 7:**
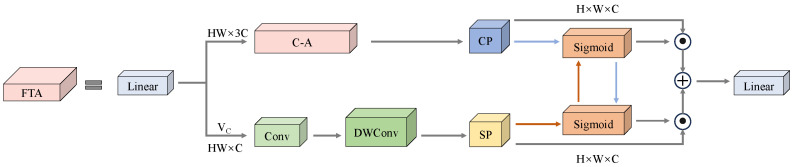
Structure of Feature Transposed Attention (FTA).

FTA differs from traditional spatial attention in that it performs self-attention mechanism calculations along the channel dimension. We divide the feature channels into multiple heads and apply channel attention, as shown in Figure 7. Let the input be X; then, the calculation of the query, key, and value (Q, K, V) in the attention mechanism process is expressed as Equation (2):

(2) $$Y_{C-A}=\mathrm{SoftMax}\left({\left(Q_{C}\right)}^{T}K_{C}/\alpha \right)\cdot V_{C},$$

where α is a learnable temperature parameter used to adjust the dot product. To extract spatial and channel features, we use $Y_{C-A}$ and $V_{C}$ for multi-feature extraction. This includes spatial projection (SP) and channel projection (CP) modules. First, we fuse global and local features on $V_{C}$ using a series of 1 × 1 convolutions and depth convolutions to obtain the preliminary channel feature $Y_{C}$. Subsequently, we perform multi-dimensional mapping on the features of the spatial and channel dimensions, as shown in Equation (3):

(3) $$Y_{S}=f_{SP}\left(Y_{C}\right),Y_{C1}=f_{CP}\left(Y_{C-A}\right),Y_{C2}=f_{CD}\left(Y_{C-A}\right),$$

where $Y_{S}$ represents the output after spatial mapping, $f_{SP}\left(\cdot \right)$ represents spatial mapping, and $f_{CP}\left(\cdot \right)$ represents channel mapping. We use 1 × 1 convolutions and deep convolutions to extract spatial information from $Y_{C}$, followed by channel segmentation. To learn more complex spatial features, we introduce nonlinear relationships on general channels. Subsequently, we perform fusion along the channel dimension to complete the spatial feature mapping. $Y_{C1}$ and $Y_{C2}$ are two outputs of channel mapping. $Y_{C1}$ further enhances $Y_{C-A}$, while $Y_{C2}$ focuses more on feature extraction and mapping in the channel dimension.

Finally, by reshaping the connections between different attentions, we obtain the channel attention feature $Y_{FTA}$. To reduce computational overhead, we only fuse spatial and channel features in the channel attention. For other feature dimensions, we use projections to obtain $Y_{S}$, $Y_{C1}$, and $Y_{C2}$, and then we perform information extraction and cross-weight transfer. The procedure is formulated in Equation (4):

(4) $$Y_{FTA}=Y_{C1}\cdot f\left(Y_{S}\right)+DWConv\left(V_{C}\right)\cdot f\left(Y_{C2}\right),$$

where f(·) denotes the sigmoid function. In the final stage, spatial features are employed to weigh the channel attention’s output, while spatial information is simultaneously utilized to perform cross-weighting on channel feature extraction. This complementary strategy achieves effective integration of spatial and channel-wise information.

**Figure 8:**
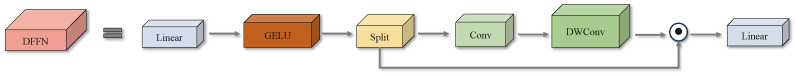
Structure of Dual-Frequency Aggregation Feedforward Network (DFFN).

To achieve selective fusion of frequency-domain information, we propose the Dual-Frequency Aggregation Feedforward Network (DFFN), as shown in Figure 8. The input $Y_{FTA}$ is first projected to $X_{in}$ through a fully connected layer, followed by a GELU activation function. A frequency gate fg is then applied to extract frequency-domain information. The frequency gate is a submodule containing two convolutional layers that decompose the input features into low-frequency and high-frequency components. The low-frequency components remain unchanged, while the high-frequency components are enhanced through 1 × 1 convolutions and depthwise convolutions (DWConv) to improve detail representation, as shown in Equation (5):

(5) $$X_{fg}=X_{in}\cdot DWConv\left(Conv_{1\times 1}\left(X_{in}\right)\right),$$

where $X_{fg}$ represents the element-wise product of these two components, producing a feature map that combines low-frequency and high-frequency information. Finally, another fully connected layer maps the fused feature map back to the original feature space $X_{DFFN}$. Through this design, the DFFN can extract frequency-domain information while preserving spatial-domain information.

### 3.4. High-Performance Multi-Scale Feature Pyramid Network (HPMSFPN)

The original YOLO11 model uses the PAN-FPN structure for feature fusion, but the PAN-FPN structure has limitations when handling multi-scale targets, especially when dealing with small targets [37]. Traditional networks rely too heavily on convolutional operations with limited receptive fields, which results in incomplete capture of information about small objects. Although methods like BiFPN [38] improve feature representation through weighted feature fusion, they still struggle to effectively balance local information with high-level semantic features, leading to poor performance in detecting small objects. To address these issues, we propose HPMSFPN, which reduces computational costs while enhancing the model’s capability to detect multi-sized residual film plastic targets.

**Figure 9:**
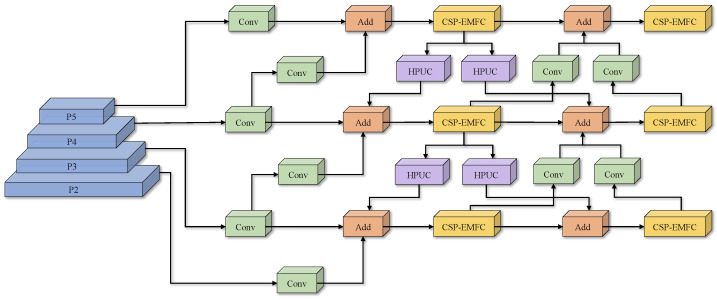
Structure of High-Performance Multi-Scale Feature Pyramid Network (HPMSFPN).

The HPMSFPN architecture is illustrated in Figure 9. HPMSFPN utilizes a cross-layer feature integration mechanism to effectively combine deep semantic representations with shallow detail characteristics and high-resolution surface-layer features, thereby achieving effective retention of feature information. For example, the Add module in the P4 layer of HPMSFPN not only fuses the P4 features at the same level and the P5 features obtained through upsampling but also fuses the P3 layer features processed through downsampling. Therefore, the Add module achieves a dual mechanism of same-scale feature fusion and cross-scale feature fusion between layers with different resolutions. HPMSFPN fully utilizes multi-scale information from different resolution layers through the above methods, effectively improving the detection accuracy of the model. Additionally, HPMSFPN replaces the original channel concatenation operation Contact with the Add operation, reducing parameter size and computational complexity while enabling adaptive selection based on the importance of features at different scales for weighted fusion.

HPMSFPN introduces efficient multi-scale feature convolution (EMFC) into the CSP, forming the CSP-EMFC module, while also adopting high-performance upsampling convolution (HPUC). These two modules are used to improve the original FPN. Subsequent sections elaborate on these enhancements in detail.

#### 3.4.1. Efficient Multi-Scale Feature Convolution (EMFC)

**Figure 10:**
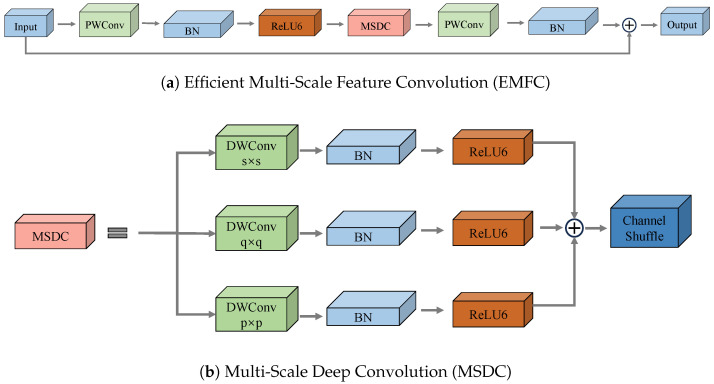
Structure of Efficient Multi-Scale Feature Convolution (EMFC) and Multi-Scale Deep Convolution (MSDC).

We propose an efficient multi-scale feature convolution module to enhance the feature representations generated by the cascaded expansion path. The EMFC module is applied to the CSP structure to form the CSP-EMFC module, as shown in Figure 5, further enhancing the model’s feature learning and fusion capabilities. The design of the EMFC module draws inspiration from the architecture of the inverted residual block (IRB) in MobileNetV2 [39]. However, unlike the IRB, the EMFC module performs deep convolution operations at different scale levels and uses channel mixing to re-combine channels across groups. The structure of EMFC is shown in Figure 10a.

Specifically, in the EMFC module, we first use a 1 × 1 pointwise convolution layer PWConv to expand the channel dimension, followed by a batch normalization layer BN and a ReLU6 activation layer. Next, we use Multi-Scale Deep Convolution (MSDC) to obtain contextual information at different scales and resolutions. Its structure is shown in Figure 10b. Since deep convolution cannot establish inter-channel correlations, we use a channel mixing operation to fuse inter-channel relationships. Subsequently, we use another pointwise convolution layer PWConv followed by a BN layer to restore the initial channel dimension while also encoding inter-channel dependencies. The computational process is illustrated in Equation (6):

(6) $$EMFC\left(x\right)=BN(PWC_{2}\left(CM\left(MSDC\left(ReLU6\left(BN\left(PWC_{1}\left(x\right)\right)\right)\right)\right)\right)),$$

where $PWC_{1}\left(\cdot \right)$ and $PWC_{2}\left(\cdot \right)$ denote two different pointwise convolutions, CM(·) denotes channel mixing, BN(·) and ReLU6(·) denote batch normalization and activation operations, respectively. The MSDC with different kernel sizes (ks) can be expressed by Equation (7):

(7) $$MSDC\left(x\right)=\sum_{ks\in KS}DWCB_{ks}\left(x\right),$$

(8) $$DWCB_{ks}\left(x\right)=ReLU6\left(BN(DWC_{ks}\left(x\right))\right),$$

where $DWC_{ks}\left(\cdot \right)$ denotes a depthwise separable convolution with kernel size ks. MSDC(·) uses recursive updates of the input *x*, where the input *x* is connected to the previous $DWCB_{ks}\left(\cdot \right)$ via a residual connection to achieve better regularization, as shown in Equation (9):

(9) $$x=x+DWCB_{ks}\left(x\right),$$

#### 3.4.2. High-Performance Upsample Convolution (HPUC)

**Figure 11:**
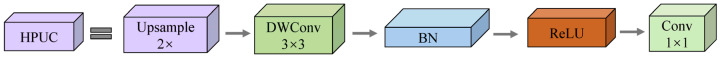
Structure of High-Performance Upsample Convolution (HPUC).

To ensure that the feature maps of the current stage match those of the next skip-connected layer in terms of dimension and resolution, we designed a High-Performance Upsample Convolution (HPUC) to achieve progressive upsampling. HPUC utilizes efficient up-convolution blocks to implement a hierarchical upsampling mechanism for the current stage’s feature map. By adjusting the dimension and resolution of the current stage’s feature map to match the specifications of the subsequent connected layer’s feature map, this process effectively promotes feature information fusion across layers and stages. The structure of HPUC is shown in Figure 11. Specifically, first, the feature representation is enlarged through an upsampling layer with a scale factor of 2. Then, a 3 × 3 depth-separable convolution enhances the characteristics within the upsampled representation. Next, batch normalization and activation function operations are performed separately. Finally, a 1 × 1 convolution adjusts channel dimensions to align with subsequent stages. This computational procedure is expressed in Equation (10):

(10) $$HPUC\left(x\right)=Conv_{1\times 1}\left(ReLU\left(BN\left(DWC\left(Up\left(x\right)\right)\right)\right)\right),$$

where Up(·) denotes an upsampling layer with a scale factor of 2, DWC(·) is a 3 × 3 depthwise separable convolution; BN(·) and ReLU(·) represent batch normalization and activation functions, respectively, and $Conv_{1\times 1}\left(\cdot \right)$ is a 1 × 1 convolution.

### 3.5. Dynamic Detect Head with DCNv4 (DWD4)

During agricultural residual film detection tasks, target shapes and dimensions exhibit significant variations. The YOLO11 struggles to deliver satisfactory performance since residual films within agricultural imagery demonstrate varying aspect ratios and irregular geometries, demanding enhanced multi-scale feature learning capabilities from the model. To effectively address scale diversity arising from varying target geometries and establish spatial correlations across multiple scales and configurations, we draw inspiration from DyHead [40], proposing the Dynamic Detect Head With DCNv4, empowering the model to successfully recognize residual plastic films with diverse morphologies.

**Figure 12:**
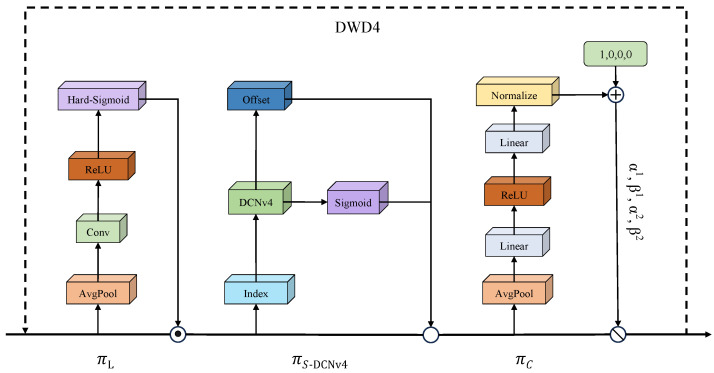
Structure of Dynamic Detect Head With DCNv4 (DWD4).

The Dynamic Detect Head With DCNv4 significantly improves the representation capabilities of the object detection head by integrating multi-head self-attention mechanisms, spatial awareness of positional information, and task-oriented perception of output channel characteristics. The key components of the Dynamic Detect Head With DCNv4 include three core attention modules: scale perception attention, spatial perception attention, and task perception attention. Scale perception attention is responsible for capturing multi-scale feature information, spatial perception attention focuses on feature extraction of spatial position information, and task perception attention focuses on semantic associations between channels. By hierarchically stacking and fusing these three types of attention mechanisms, it is possible to achieve more comprehensive feature capture of residual membrane target features, resulting in more accurate detection results. The structure of DWD4 is shown in Figure 12.

In the spatial perception attention module, Deformable convnets v2 (DCNv2) [41] brings extra computational burden during non-adjacent location sampling, leading to delayed convergence. Therefore, our approach enhances the deformable convolution within the architecture by substituting the original DCNv2 with Deformable convnets v4 (DCNv4) [42]. DCNv4 is an efficient dynamic sparse operator that enhances the network’s dynamic adaptability and representational capabilities by removing the softmax normalization operation during spatial aggregation. This design addresses the inherent limitations of softmax, namely convergence speed constraints and insufficient operator expressiveness. DCNv4 introduces an adaptive aggregation window and employs a dynamic unconstrained weighting strategy, thereby achieving greater flexibility and adaptability in feature processing. In terms of computational optimization, DCNv4 optimizes memory access patterns using instruction-level kernel analysis technology, effectively reducing redundant operations and significantly improving computational efficiency. The Dynamic Detect Head With DCNv4 calculation process is shown in Equation (11):

(11) $$W\left(F\right)=\pi_{C}\left(\pi_{S-DCNv4}\left(\pi_{L}\left(F\right)\cdot F\right)\cdot F\right)\cdot F,$$

where $F\in R^{L\times S\times C}$ is a feature tensor with three dimensions. *L* denotes the feature map hierarchy, *S* corresponds to the spatial dimension product (width × height) of the feature map, and *C* indicates the channel count of the feature map. $\pi_{L}\left(\cdot \right)$, $\pi_{S-DCNv4}\left(\cdot \right)$, and $\pi_{C}\left(\cdot \right)$ represent the three attention functions acting on *L*, *S*, and *C*, respectively. $\pi_{L}\left(\cdot \right)$ is the scale perception attention function, $\pi_{S-DCNv4}\left(\cdot \right)$ is the spatial perception attention function, and $\pi_{C}\left(\cdot \right)$ is the task perception attention function.

Three modules are stacked in sequence. First, the scale attention module $\pi_{L}\left(\cdot \right)$ dynamically fuses features based on the semantic importance of different scales. Its calculation formula is shown in Equation (12):

(12) $$\pi_{L}\left(F\right)\cdot F=\sigma \left(f\left(\frac{1}{SC}\sum_{S,C}F\right)\right)\cdot F,$$

where f(·) represents a linear function that is approximated through a 1 × 1 convolutional layer, and $\sigma \left(\cdot \right)=\max(0,\min\left(1,\frac{x+1}{2}\right))$ denotes the hard-sigmoid activation function.

Next is the spatial perception attention module utilizing the fused features, which concentrates on distinctive regions that persistently exist across both spatial coordinates and feature hierarchies. This module uses a two-stage method, initially inducing sparsity in attention learning through deformable convolution, followed by feature aggregation across different levels at identical spatial coordinates. The calculation formula is shown in Equation (13):

(13) $$\pi_{S-DCN\nu 4}\left(F\right)\cdot F=\frac{1}{L}\sum_{l=1}^{L}\sum_{k=1}^{K}w_{l,k}\cdot F\left(l;p_{k}+\Delta p_{k};c\right)\cdot \Delta m_{k},$$

where *K* represents the count of sparse sampling positions, $p_{k}+\Delta p_{k}$ denotes a displaced location through the self-adaptive spatial displacement $p_{k}$ to concentrate on a discriminative area, and $\Delta m_{k}$ constitutes a self-adaptive importance weight at position $p_{k}$. Both parameters are derived from the input features at the intermediate level of F.

To facilitate collaborative learning and accommodate diverse object representations, we implement a task perception attention mechanism as the concluding module. This mechanism adaptively activates and deactivates feature channels according to task-specific requirements. The formula for calculating task perception attention is shown in Equation (14):

(14) $$\pi_{C}\left(F\right)\cdot F=\max\left(\alpha^{1}\left(F\right)\cdot F_{c}+\beta^{1}\left(F\right),\alpha^{2}\left(F\right)\cdot F_{c}+\beta^{2}\left(F\right)\right),$$

where $F_{c}$ denotes the feature slice of channel c, and ${\left[\alpha^{1},\alpha^{2},\beta^{1},\beta^{2}\right]}^{T}=\theta \left(\cdot \right)$ constitutes a hyperfunction responsible for learning and controlling the activation threshold parameters.

Ultimately, given that the aforementioned three attention mechanisms are implemented in sequence, we can recursively apply Equation (11) to efficiently combine multiple $\pi_{L}$, $\pi_{S-DCNv4}$, and $\pi_{C}$ modules in a stacked configuration to form the Dynamic Detect Head With DCNv4.

**Figure 13:**
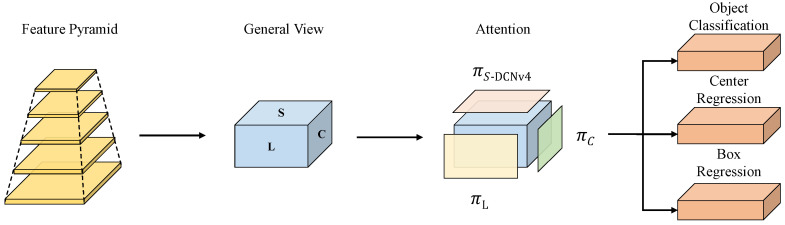
Illustration of the Dynamic Detect Head With DCNv4 (DWD4).

As shown in Figure 13, the working principle of the Dynamic Detect Head With DCNv4 is as follows: First, the feature map passes through the scale perception attention module, making it more sensitive to multi-scale differences in residual film targets. Then, through spatial perception attention module, it utilizes the adaptive convolution mechanism and sparse connection characteristics of DCNv4 to achieve adaptive focusing of feature maps on the discriminative spatial positions of foreground objects, effectively addressing the challenge of scale changes in residual film target detection while improving computational efficiency. Finally, the task perception attention module adjusts feature representations according to the specific demands of distinct tasks, generating task-oriented feature activation patterns to optimize the model’s task-specific representation capabilities.

## 4. Experiments

### 4.1. Experimental Environment

The experiments in this study were conducted on a server running the Ubuntu operating system with CUDA Vision 11.8, equipped with an NVIDIA GeForce RTX 4090 graphics card (manufactured by NVIDIA Corporation in Santa Clara, CA, USA) featuring 24 GB of video memory. The programming language and deep learning frameworks employed were Python 3.8, nvcc 11.0, PyTorch 2.2.0, and torchvision 0.9.0. The experimental hyperparameters were as follows: an initial learning rate of 0.01 with a cosine annealing learning rate scheduler, input image size of 640 × 640, 300 epochs, and a batch size of 16. The Stochastic Gradient Descent (SGD) optimizer was employed with a weight decay of 0.0005 and momentum of 0.937. The model utilized a combination of classification loss (Binary Cross-Entropy), objectness loss (Binary Cross-Entropy), and box regression loss (Complete Intersection over Union loss) following the standard YOLO11 multi-task loss function.

The experimental data consists of a self-collected agricultural film collection, which includes imagery of plastic films acquired from field environments with manual annotation. This collection encompasses 1900 agricultural film samples, distributed according to an 8:1:1 split, producing 1520 training samples, 190 validation samples, and 190 testing samples. Online data augmentation strategies were applied to enhance the residual film data. The online augmentation operates in real time during training with a 0.5 probability, applying geometric transformations, mosaic and mixup operations, and noise injection while maintaining annotation accuracy, thereby maximizing data diversity and preventing overfitting without additional storage requirements.

### 4.2. Evaluation Metrics

This study employs precision (P), recall (R), mean average precision (mAP), and F1 score as evaluation metrics to assess the detection performance of the proposed model. Precision quantifies the fraction of correctly identified positive samples among all samples classified as positive, while recall measures the fraction of true positive instances successfully detected by the model. Enhanced precision indicates a superior percentage of accurate residual film target detections, whereas improved recall demonstrates the model’s capability to identify a greater proportion of actual residual film targets. The F1 score represents the harmonic mean of precision and recall, providing equal weighting to both metrics [43]. The formulas are as follows:

(15) $$\mathrm{Precision}=\frac{\mathrm{TP}}{\mathrm{TP}+\mathrm{FP}},$$

(16) $$\mathrm{Recall}=\frac{\mathrm{TP}}{\mathrm{TP}+\mathrm{FN}},$$

(17) $$F1=\frac{2\times \mathrm{Precision}\times \mathrm{Recall}}{\mathrm{Precision}+\mathrm{Recall}},$$

where TP denotes the number of true positive samples, representing instances correctly classified as positive when they are indeed positive. FP represents the number of false positive samples, corresponding to instances incorrectly classified as positive when they are actually negative. FN indicates the number of false negative samples, referring to instances incorrectly classified as negative when they are actually positive.

Average precision (AP) is computed through integration of the area under the precision–recall (P-R) curve. The mAP represents the mean AP across all object categories, serving as a comprehensive indicator of overall detection accuracy. Superior mAP values indicate enhanced model performance across diverse object categories. Following the COCO evaluation metrics, two Intersection over Union (IoU) thresholds were employed, 0.5 and 0.5 0.95:0.05 (where 0.05 denotes the step size), yielding mAP50 and mAP50-95 metrics to evaluate model detection performance under varying strictness conditions. The calculation formulas are as follows:

(18) $$\mathrm{AP}=\int_{0}^{1}P\left(r\right)\mathrm{dr},$$

(19) $$\mathrm{mAP}=\frac{\sum_{i}^{K}AP_{i}}{K},$$

where P(r) represents the precision value associated with recall R along the precision–recall curve, and K indicates the total count of object classes.

Computational complexity of the model is evaluated using FLOPs and model size as primary metrics. FLOPs quantify the total number of floating-point operations required by the model during inference. Elevated FLOP counts indicate increased computational complexity, necessitating greater computational resources for model execution. Model size typically correlates with architectural complexity, requiring proportionally larger storage capacity for deployment.

Detection speed performance is assessed through FPS and inference time measurements. FPS represents the throughput capacity of the target network, specifically the number of images processed per second. Higher FPS values indicate reduced computational overhead and enhanced processing efficiency. Conversely, extended inference times suggest elevated computational complexity, requiring a prolonged processing duration for input data analysis.

### 4.3. Ablation Experiments

To better validate the effectiveness of the modules, we conducted combination experiments with different modules on the residual membrane dataset. In the experiments, we used the original YOLO11s model as the baseline and gradually added the Freq-C3 module, HPMSFPN, and DDW4 to perform ablation experiments, comparing the performance metrics of different models. The experimental results are shown in Table 1. As can be seen from the table, after adding the Freq-C3 module to the model, the mAP50 improved by 4.15% relative to baseline. This demonstrates that the model achieves enhanced perception of residual film targets in soil backgrounds through dynamic calibration via attention mechanisms and frequency-domain feature enhancement, effectively addressing the challenge of identifying residual film targets with low contrast in agricultural residue detection tasks. After incorporating HPMSFPN into the model, the mAP50 improved by 1.21%, indicating that the model achieves effective fusion of multi-scale features through a cross-level feature fusion strategy, significantly enhancing detection accuracy for residual film targets of different sizes. Similarly, after adding DDW4 to the model, the mAP50 improved by 1.06%, indicating that the model can adaptively handle irregular shape changes of residual film by fusing multi-head self-attention mechanisms and adopting an improved DCNv4, effectively addressing the issue of arbitrary deformation of residual film in the task of detecting residual film in farmland. When the three modules are used together, the mAP50 improves by 5.37%, reaching 79.53%, achieving excellent results. Experiments show that these three modules are independent of each other and do not cause a decrease in accuracy due to constraints. By efficiently fusing residual features under multi-scale receptive fields, integrating shallow-layer detail features with deep-layer semantic information, the model achieves precise identification of residual film targets.

To validate the universality of the proposed modules, we conducted comprehensive ablation experiments on the YOLO12 model, with results presented in Table 2. The experimental data reveal that when individually integrating the FreqC3, HPMSFPN, and DWD4 modules, the model achieves mAP50 scores of 76.11%, 75.13%, and 76.25%, respectively, all demonstrating varying degrees of performance improvement compared to the baseline YOLO12 model. More significantly, when all three modules work synergistically, the mAP50 value further increases to 76.67%, representing a substantial improvement of 1.68% over the original YOLO12 model. These experimental results conclusively demonstrate that our proposed modules not only excel in YOLO11 but also achieve superior performance across different model architectures, such as YOLO12, showcasing excellent cross-architectural generalization capability and synergistic effects among the modules.

### 4.4. Comparison Experiments

To assess FreqDyn-YOLO’s detection capabilities, we performed comparative analysis against established object detection architectures and contemporary YOLO variants, encompassing Faster R-CNN, YOLOv3, YOLOv5, YOLOv8s, YOLO11n, YOLO11s, YOLO11x, and YOLO12s. All models were trained on the manually annotated residual membrane dataset under identical configuration environments. To ensure experimental reliability, each model was trained three times, with the optimal results selected as comparison metrics.

The detection performance comparison across different models is presented in Table 3, while the computational complexity comparison is shown in Table 4. Analysis reveals that Faster R-CNN, representing traditional two-stage detection architectures, exhibits considerably reduced mAP50 relative to prevalent single-stage YOLO frameworks. YOLOv3, an established representative within YOLO variants, exhibits a 12.09% better mAP50 than Faster R-CNN but falls 2.62% short of YOLO11s’s performance. YOLOv5 shows a 2.57% improvement over YOLOv3, though it underperforms compared to YOLOv8, YOLO11n, YOLO11x, and YOLO12s.

Among the compared models, YOLO11n exhibits the lowest computational complexity but achieves only 73.41% mAP50. YOLO11x attains the highest mAP50 of 75.42%, representing a 2.01% improvement over YOLO11n; however, its computational complexity of 194.4 FLOPs makes it unsuitable for hardware deployment. YOLO11s demonstrates balanced performance across precision, recall, mAP, and computational complexity metrics. The proposed FreqDyn-YOLO model, incorporating the FreqC3, HPMSFPN, and DDW4 modules, enhances feature extraction capabilities for residual films. Relative to the baseline approach, performance gains of 5.37%, 1.97%, and 2.96% are observed for mAP50, precision, and recall, respectively. Additionally, FPS increased from 44.71 to 77.69. When compared to YOLO12s, the proposed model demonstrates superior performance in precision, F1 score, mAP, and detection speed. FreqDyn-YOLO12s represents the implementation of our three proposed modules integrated into the YOLO12s. While this variant demonstrates notable performance improvements compared to the baseline YOLO12s model, comprehensive evaluation reveals that it exhibits inferior performance across multiple key metrics when benchmarked against FreqDyn-YOLO, including reduced precision, recall rate, F1 score, mAP50, and slower detection speed. This comparative analysis underscores the architectural advantages of the YOLO11-based FreqDyn-YOLO implementation. The FreqDyn-YOLO model achieves both high detection accuracy and fast processing speed, satisfying practical deployment demands.

### 4.5. FreqC3 Position Combination

YOLO11 enables interaction between shallow and deep features through cross-layer connections of the C3k2 module, combined with upsampling and downsampling operations, providing enhanced feature information for subsequent multi-scale object detection branches. The backbone of YOLO11 contains four C3k2 modules, located at four different layers: P2, P3, P4, and P5. The P2 layer processes the highest resolution feature maps to extract fine-grained detail features for improved small-object detection. The P3, P4, and P5 layers handle progressively lower resolution feature maps, targeting small-, medium-, and large-object detection, respectively.

To assess FreqC3’s performance contribution, we maintained the FreqC3 module consistently at the P2 layer and systematically added FreqC3 modules to the P3, P4, and P5 layers through various combinations. Layers not utilizing the FreqC3 module retained the original C3k2 structure. The experimental results are presented in Table 5.

The results demonstrate that the model’s mAP increases proportionally with the number of FreqC3 modules implemented. When the FreqC3 module is maintained at the P2 layer and a single additional FreqC3 module is introduced, the highest mAP50 of 75.61% is achieved when applied to the P5 layer. Using FreqC3 modules at both the P3 and P4 layers yields an mAP50 of 76.15%. Complete replacement of all C3k2 modules with FreqC3 modules produces the highest experimental mAP50 of 78.35%.

The experimental findings confirm that each FreqC3 module addition contributes positively to performance. The FreqFTA mechanism improves the model’s ability to distinguish subtle differences between residual films and background elements, while the DFFN component significantly enhances surface texture feature extraction capabilities for residual films, thereby confirming FreqC3’s superior performance.

### 4.6. EMFC Size Selection

In HPMSFPN, EMFCs of different sizes are used to extract features from depth features, resulting in feature maps of different scales. To better validate the impact of using convolutional kernels of different sizes for feature selection on feature maps of different scales, ablation experiments were conducted using convolutional kernels of different sizes in the P3, P4, and P5 stages, as detailed in Table 6. As shown in the table, when uniformly using small convolutional kernels of sizes (1, 3, 5) across all layers, P3, P4, and P5, the recognition precision was 74.52%, with the lowest recall of 65.29%. This indicates that uniformly using small convolutional kernels results in a significant number of residual membrane targets not being identified. In contrast, uniformly using convolutional kernels of sizes (3, 5, 7), which are medium-sized convolutional kernels, yields the opposite result, with a higher recall of 67.46% but a lower precision of 74.15%. This shows that while medium-sized convolutional kernels can better capture residual membrane targets, the increased number of false positives generates many invalid prediction boxes, leading to lower accuracy. When uniformly using large convolutional kernels of sizes (5, 7, 9), both the issues associated with small and medium-sized convolutional kernels arise, resulting in a precision of only 70.60% and a recall of only 65.91%, with both precision and recall decreasing. Therefore, we designed a set of experiments using kernel sizes of (1, 3, 5), (3, 5, 7), and (5, 7, 9) in layers P3, P4, and P5, respectively. The different receptive fields precisely matched different target scales, enabling the model to balance precision and recall while maintaining a high level, with an mAP50 of 75.37%. The results indicate that the hierarchical convolution kernel design can help the HPMSFPN better adaptively select feature information based on different scales.

### 4.7. Deformable Convolution Version Selection

The spatial perception attention module of the Dynamic Detect Head employs deformable convolutions to implement a sparse attention learning mechanism. To evaluate the impact of different deformable convolution versions on model performance, this study integrated DCNv2, DCNv3, and DCNv4 into the spatial perception attention module for comparative experiments, with results presented in Table 7. Analysis reveals that DCNv2 exhibits certain limitations in detection tasks. Its recall is only 64.60%, and mAP50 reaches 72.96%. Although DCNv2 has relatively low computational complexity, its adaptive capability when processing irregularly shaped targets is limited, and its inference speed is relatively low. DCNv3 shows significant improvements over DCNv2 in multiple metrics. The recall increased to 70.86%, a relative improvement of 6.26%, and the mAP50 increased to 75.06%, a relative improvement of 2.10%. However, this performance improvement comes with increased computational overhead, indicating that DCNv3 enhances detection performance for complex-shaped objects through improved feature learning capabilities. DCNv4 demonstrates superior performance across all evaluation dimensions. This version records 72.48% precision and 70.38% recall, with mAP50 reaching 75.22%, all of which are the highest values among the three versions. Additionally, DCNv4 performs exceptionally well in computational efficiency, with a computational complexity of 22.1 GFLOPs and an inference speed of 66.56 FPS, achieving the optimal balance between accuracy and efficiency. Experimental results indicate that DCNv4 possesses stronger adaptive sampling capabilities and feature representation capabilities, dynamically adjusting the sampling positions of convolutional kernels based on the spatial distribution of input features to more effectively capture the geometric features of irregular objects. Compared to DCNv2 and DCNv3, DCNv4 significantly improves detection accuracy while maintaining computational efficiency, providing the optimal technical choice for the spatial perception attention module of dynamic detection heads.

### 4.8. Visualization Experiments

**Figure 14:**
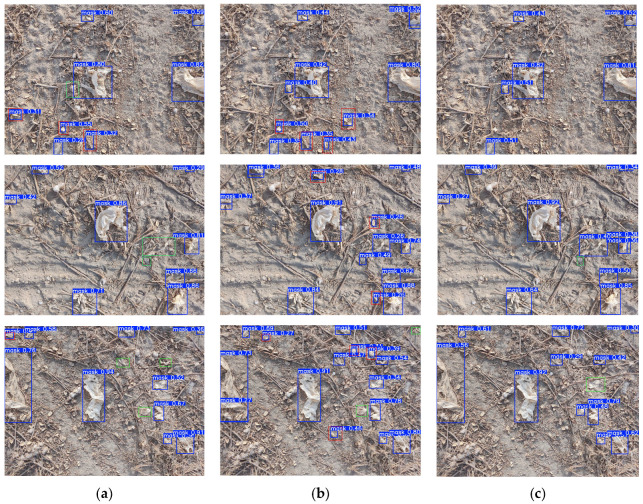
Visualization of detection results from different models. (**a**) YOLO11. (**b**) YOLO12. (**c**) FreqDyn-YOLO.

Visualization results of residual film images using the YOLO11 model, the latest YOLO12 model, and the improved FreqDyn-YOLO model are shown in Figure 14. Red boxes indicate detection errors, while green boxes indicate undetected areas. In the first row, both YOLO11 and YOLO12 incorrectly detected dry leaves or cotton as residual films, and YOLO11 also failed to detect some residual films. In contrast, FreqDyn-YOLO showed no issues with either false detections or missed detections, demonstrating significantly superior detection performance compared to the other models. In the second row, both YOLO11 and FreqDyn-YOLO have missed detection issues, but FreqDyn-YOLO has fewer missed detections, and it does not have any false detection issues. YOLO12 does not have any missed residual film detections but has numerous false detections. In the last row, while all three models experienced missed detections, FreqDyn-YOLO demonstrated superior performance compared to the other two by avoiding false detections entirely and achieving fewer missed detections, further confirming that the FreqDyn-YOLO model has better detection performance than other models.

**Figure 15:**
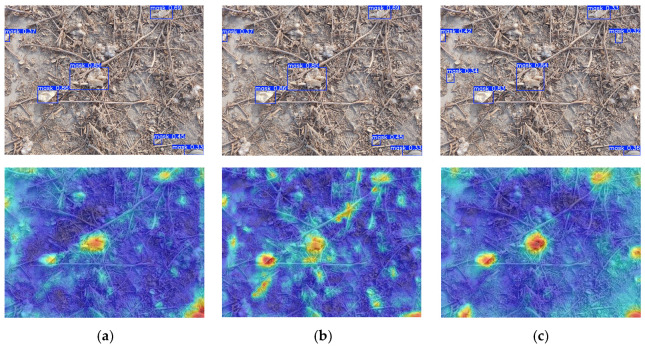
Heatmap visualization of detection results from different models. (**a**) YOLO11. (**b**) YOLO12. (**c**) FreqDyn-YOLO.

Heatmaps are used to visualize the detection results of YOLO11, YOLO12, and FreqDyn-YOLO, as shown in Figure 15. The activation distribution of the YOLO11 model falls between that of YOLO12 and FreqDyn-YOLO. While there is a clear response in the target area, some residual film targets do not exhibit high-intensity activation responses. Additionally, the clarity and intensity contrast of the activation boundaries are inferior to those of FreqDyn-YOLO. The activation pattern of the YOLO12 model is relatively dispersed, with a significant number of moderate-intensity activation responses (green regions) appearing in non-residual film target areas, indicating that the model has certain limitations in distinguishing between residual film and soil backgrounds. In contrast, the improved FreqDyn-YOLO model exhibits the most precise activation response pattern, with high activation regions (red/orange) in the heatmap precisely focused on residual film target locations. The activation intensity distribution shows a distinct gradient decay characteristic, decreasing gradually from the target center toward the edges. This feature indicates that the FreqFTA mechanism in the FreqC3 feature extraction module effectively enhances the model’s perception of residual film boundaries. In the residual film heatmap of FreqDyn-YOLO, the soil area maintains a stable low activation level (dark blue), which is attributed to the enhanced perception of residual film surface texture features by the frequency gating mechanism of DFFN. In contrast, YOLO11 and YOLO12 exhibit more noise activation in the background area, indicating relatively weaker feature discrimination performance. As shown by the activation regions of different residual film targets in the heatmap, FreqDyn-YOLO also demonstrates more balanced multi-scale detection capabilities, achieving corresponding activation intensities for both large-area and small-sized residual film targets. This is because the dual-feature fusion mechanism implemented by the EMFC and HPUC modules in HPMSFPN effectively overcomes the limitations of traditional feature pyramid networks in small target detection. Furthermore, the heatmap of FreqDyn-YOLO exhibits clearer spatial definition at the edges of residual films, with the highest alignment between activation regions and target contours. This improvement is primarily attributed to the application of DCNv4 in the DWD4 dynamic detection head, which significantly enhances the network’s adaptability to complex-shaped residual film targets through dynamic unconstrained weighting strategies and adaptive aggregation windows.

## 5. Discussion

The experimental results demonstrate that FreqDyn-YOLO achieves superior performance compared to existing YOLO versions and traditional detection frameworks. Notably, while Faster R-CNN exhibits longer inference times as expected, the performance gap is not as substantial as anticipated; moreover, it fails to achieve better detection accuracy even when compared to older YOLO versions.

From multiple research perspectives, YOLO and Faster R-CNN demonstrate distinct advantages and limitations in object detection tasks. YOLO achieves end-to-end optimization and exceptionally high inference speed by treating object detection as a regression problem, directly predicting bounding boxes and class probabilities from complete images. In contrast, while Faster R-CNN typically exhibits superior accuracy performance, it suffers from longer inference times, particularly when processing complex scenes, where its speed and efficiency may be inferior to YOLO-based approaches [44,45].

FreqDyn-YOLO faces numerous challenges when applied to more complex agricultural environments and larger-scale agricultural film residue detection. Soil types, lighting conditions, film residue materials, and aging degrees vary significantly across different geographical regions, and these factors may affect the generalization ability of the Frequency Feature Transposed Attention mechanism in processing frequency-domain information. Especially under extreme weather conditions or complex background interference, attention calculations in the channel dimension may require a larger training sample size to maintain detection accuracy stability.

The Dual-Frequency Aggregation Feedforward Network (DFFN) achieves selective fusion of frequency-domain information through a frequency gating mechanism, but the adaptive adjustment of frequency gating thresholds when faced with diverse residual film texture features still requires further optimization. Residual film in different agricultural regions may exhibit shifts in frequency-domain feature distributions, posing new challenges for the model’s cross-domain adaptability. Additionally, the cross-level feature fusion mechanism in the High-Performance Multi-Scale Feature Pyramid Network (HPMSFPN) may incur excessive computational overhead when processing large-scale images, particularly when deployed on resource-constrained agricultural equipment.

While FreqDyn-YOLO demonstrates improved detection performance compared to existing approaches, the integration of specialized modules such as FreqC3, DCNv4, and EMFC introduces additional computational complexity. The operations in FreqC3 and deformable convolutions in DCNv4 require substantial memory allocation and processing power, which may present challenges for deployment on embedded systems commonly used in agricultural machinery. The performance characteristics on edge computing platforms with limited resources remain to be comprehensively evaluated to ensure practical viability in field operations.

FreqDyn-YOLO can be integrated with residual film recovery machines for application in the collection process. By combining the model’s detection results with GPS positioning systems and path planning algorithms, the detected residual film location information can be converted into control instructions for the film retrieval device, enabling full-process automation from detection to recovery. This significantly improves residual film recovery efficiency and reduces labor costs. However, the successful implementation of such integrated systems requires consideration of environmental factors typical in agricultural settings, including dust accumulation, mechanical vibrations, temperature variations, and intermittent connectivity issues that may affect system reliability and performance consistency.

Successful deployment requires addressing several critical limitations. Future research should focus on the following: developing lightweight versions through model pruning, knowledge distillation, and quantization techniques for edge computing devices; conducting comprehensive performance evaluations on typical agricultural embedded platforms including inference latency, memory usage, and power consumption analysis; establishing datasets covering diverse agricultural regions and conditions to improve cross-domain adaptability; and performing long-term field trials with agricultural cooperatives to validate practical effectiveness and economic viability under real farming conditions, including dust, vibration, temperature fluctuations, and unstable network connectivity.

## 6. Conclusions

Accurate identification of residual film targets is essential for effective agricultural management and environmental conservation. This study addresses the critical challenge of detecting residual plastic mulch in agricultural environments by proposing FreqDyn-YOLO, an improved object detection model based on the YOLO11 architecture. The research makes three significant technical contributions to overcome the difficulties posed by the visual similarity between plastic film remnants and soil backgrounds, as well as the morphological diversity of target objects.

First, we introduce the FreqC3 feature extraction module, incorporating the Frequency Feature Transposed Attention (FreqFTA) mechanism, which effectively enhances the model’s ability to discriminate between residual film and soil substrates. The FreqFTA mechanism achieves this through channel-wise attention computation and dual-frequency aggregation, significantly improving the detection of surface texture features and edge information of residual plastic film.

Second, the High-Performance Multi-Scale Feature Pyramid Network (HPMSFPN) successfully addresses the limitations of traditional PAN-FPN in small-target detection. Through cross-layer feature fusion mechanisms and the integration of EMFC and HPUC modules, HPMSFPN effectively combines semantic and detail features across different scales, enhancing detection performance for targets of varying dimensions.

Third, the Dynamic Detection Head With DCNv4 (DWD4) provides improved adaptability to the diverse morphological characteristics of residual plastic films. We replace DCNv2 with DCNv4, remove softmax normalization, and introduce adaptive aggregation windows, resulting in enhanced dynamic adaptation capabilities and computational efficiency.

To validate the effectiveness of the proposed model for residual film detection, experimental evaluations were performed using a dataset collected from natural agricultural environments. Experimental validation demonstrates that FreqDyn-YOLO achieves substantial improvements over the baseline model, with increases of 5.37%, 1.97%, and 2.96% in mAP50, precision, and recall, respectively. The model also outperforms other state-of-the-art detection methods, establishing its effectiveness for practical applications. The proposed FreqDyn-YOLO model provides a reliable technical foundation for precise identification of residual plastic films in agricultural settings. The research contributes to developing automated recovery systems for plastic mulch, supporting sustainable agricultural practices and environmental protection.

Future work may focus on expanding the dataset diversity, optimizing model deployment for real-time applications, and integrating the detection system with robotic recovery machinery for complete automation of plastic mulch management in agricultural fields.

## Figures and Tables

**Table 1:** Ablation experiments of different modules on YOLO11.

FreqC3	HPMSFPN	DDW4	P (%)	R (%)	F1 (%)	mAP50 (%)	mAP50-95 (%)
–	–	–	75.53	67.36	71.21	74.16	46.30
√	–	–	76.49	72.21	74.29	78.35	50.38
–	√	–	75.95	68.74	72.17	75.37	48.62
–	–	√	72.48	70.38	71.41	75.22	47.29
√	√	√	77.50	70.32	73.73	79.53	51.36

√ indicates the method is applied, – indicates the method is not applied.

**Table 2:** Ablation experiments of different modules on YOLO12.

FreqC3	HPMSFPN	DDW4	P (%)	R (%)	F1 (%)	mAP50 (%)	mAP50-95 (%)
–	–	–	73.32	65.58	69.23	74.99	47.68
√	–	–	74.35	70.12	72.17	76.11	49.24
–	√	–	75.62	66.70	70.88	75.13	47.48
–	–	√	71.07	71.20	71.14	76.25	47.50
√	√	√	72.58	69.18	70.84	76.67	49.25

√ indicates the method is applied, – indicates the method is not applied.

**Table 3:** Comparison of different models.

MODEL	P (%)	R (%)	F1 (%)	mAP50 (%)	mAP50-95 (%)
Faster R-CNN	58.21	44.83	50.65	59.45	29.34
YOLOv3	70.24	52.10	59.83	71.54	36.90
YOLOv5	72.63	67.81	70.14	74.11	44.60
YOLOv8s	72.72	66.13	69.27	74.23	48.21
YOLO11n	73.74	66.37	70.91	73.41	44.71
YOLO11s	75.53	67.36	71.21	74.16	46.30
YOLO11x	74.81	68.13	71.31	75.42	49.73
YOLO12s	73.32	65.58	69.23	74.99	47.68
FreqDyn-YOLO12s	72.58	69.18	70.84	76.67	49.25
FreqDyn-YOLO	77.50	70.32	73.73	79.53	51.36

**Table 4:** Model computational complexity analysis.

MODEL	FLOPs (G)	Model Size (MB)	FPS	Inference Time (ms)
Faster R-CNN	262.6	315.2	34.40	29.07
YOLOv3	135.2	234.4	40.45	24.72
YOLOv5	15.8	13.7	50.34	19.86
YOLOv8s	28.4	16.5	45.56	21.94
YOLO11n	6.3	5.4	50.64	19.74
YOLO11s	21.3	18.3	44.71	22.36
YOLO11x	194.4	109.1	35.37	28.27
YOLO12s	19.3	17.8	53.27	18.77
FreqDyn-YOLO12s	24.9	15.7	56.27	17.77
FreqDyn-YOLO	24.1	15.6	77.69	12.87

**Table 5:** FreqC3 performance at different locations.

P2	P3	P4	P5	P (%)	R (%)	F1 (%)	mAP50 (%)	mAP50-95 (%)
–	–	–	–	75.53	67.36	71.21	74.16	46.30
√	–	–	–	76.09	64.97	70.10	74.68	47.87
√	√	–	–	73.62	68.79	71.12	75.23	46.84
√	–	√	–	74.00	67.07	70.36	75.18	48.65
√	–	–	√	77.33	67.61	72.15	75.61	47.89
√	√	√	–	74.21	68.74	71.37	76.15	48.45
√	√	–	√	72.31	68.24	70.21	74.80	48.17
√	–	√	√	75.04	67.95	71.32	75.31	48.23
√	√	√	√	76.49	72.21	74.29	78.35	50.38

√ indicates the method is applied, – indicates the method is not applied.

**Table 6:** Different size selections for Efficient Multi-Scale Feature Convolution.

P3	P4	P5	P (%)	R (%)	F1 (%)	mAP50 (%)	mAP50-95 (%)
(1, 3, 5)	(1, 3, 5)	(1, 3, 5)	74.52	65.29	69.60	74.13	47.52
(3, 5, 7)	(3, 5, 7)	(3, 5, 7)	74.15	67.46	70.65	74.80	47.08
(5, 7, 9)	(5, 7, 9)	(5, 7, 9)	70.60	65.91	68.17	73.94	47.46
(1, 3, 5)	(3, 5, 7)	(5, 7, 9)	75.95	68.74	72.17	75.37	48.62

**Table 7:** Deformable convolution performance comparison.

Version	FLOPs (G)	FPS	P (%)	R (%)	F1 (%)	mAP50 (%)	mAP50-95 (%)
DCNv2	21.8	62.26	72.44	64.60	68.29	72.96	45.66
DCNv3	22.9	64.48	70.55	70.86	70.70	75.06	46.77
DCNv4	22.1	66.56	72.48	70.38	71.41	75.22	47.29

## Data Availability

Data can be made available upon request from the authors.
